# The molecular profile of the inflammatory process differs among various neurodevelopmental disorders with or without cognitive component: A hypothesis of persistent systemic dysfunction and hyper-resolution

**DOI:** 10.3389/fped.2023.1132175

**Published:** 2023-04-20

**Authors:** Miriam Madrid, Rafael Bojalil, Malinalli Brianza-Padilla, Jasbet Zapoteco-Nava, Ricardo Márquez-Velasco, Rolando Rivera-González

**Affiliations:** ^1^ Department of Health Care, Universidad Autónoma Metropolitana-Xochimilco, Mexico City, Mexico; ^2^ Department of Immunology, Instituto Nacional de Cardiología Ignacio Chávez, Mexico City, Mexico; ^3^ Neurodevelopmental Research Center, Instituto Nacional de Pediatría, Mexico City, Mexico

**Keywords:** inflammatory molecules, hyper-resolution, tissue dysfunction, regulatory disorders, cognitive impairment, neurodevelopmental disorders

## Abstract

**Introduction:**

Challenges of diverse origin in childhood can alter the growth and development of the central nervous system, affecting structures and functions. As a consequence of the damage suffered during the perinatal period, long periods of dysfunctionality may occur, such as regulatory disorders, which may result in remaining in a process of low-grade inflammation. We previously found that perinatal risks and neurological signs are associated with long-term changes in circulating concentrations of molecules of the inflammatory process, findings that are consistent with the postulate that long periods of dysfunction may condition long-lasting low-grade inflammation or parainflammation. The aim of this study was to assess whether different expressions of neurological disorders show variations in their inflammatory molecule profiles or whether there is a common pattern.

**Methods:**

We included screening for (a) caregiver-perceived risk detection of regulatory disturbances, using the DeGangi instrument; (b) dysautonomia or asymmetries, through neurodevelopmental assessments; (c) cognitive developmental disturbances (using the Bailey instrument). We assessed protein molecules on a multiplex system, and lipid molecules by ELISA.

**Results:**

We found a similar, although not identical, pattern of cytokine profiles with the presence of risk of regulatory disturbances, dysautonomia and asymmetries; but an opposite inflammatory profile was associated with cognitive impairment.

**Discussion:**

Our results suggest that there are diverse, probably limited, molecular footprints associated with impaired function, and that these footprints may depend on the response requirements necessary to adjust to the altered internal environment. Here we propose a theoretical model that suggests possible scenarios for inflammatory outcomes associated with chronic challenges.

## Introduction

Regulatory disorders, which can include multiple alterations, can be the result of different risk factors such as premature birth (1, 2) and damage during the perinatal period (3). Perinatal asphyxia in term newborns is the most important cause of brain damage and neurological sequelae, responsible for motor, sensory and cognitive disturbances, with high individual, family, and social costs (4). These disorders can be expressed as difficulties in the behavior regulation (i.e., hyper-reactive or hypo-reactive responses to environmental stimuli, such as excessive crying, aggressiveness, prolonged time to self-regulate tantrums, decreased or increased responses to pain, or poor tolerance to change of routines), vegetative alterations (regulation of heart rate, temperature, circadian rhythms, etc.), alterations in sensory processing and cognitive executive functions that could directly affect their school opportunities (5, 6). Although learning difficulties, by their nature, only manifest from school age onwards and can continue into adulthood, the cognitive functions necessary for adequate development can be studied at preschool age (7).

Neurodevelopment in children has a continuous evolution, with a critical influence by both intrinsic and extrinsic stimuli. Extrinsic stimuli organize the functions of the developing individual and stimulate intrinsic homeostasis, allowing a regulated response to the challenges of the internal and external environments. Thus, a condition that disrupts the growth and development of central nervous system structures may delay the organization of homeostatic responses such as those dependent on neuro-immune interactions. However, by going through long periods of dysfunctionality, such children remain in a process of low-grade inflammation or parainflammation, which could, in turn, condition the permanence of nervous system dysfunctions, or the presence of dysfunctions in other organs or systems (8, 9).

In a previous study, we found that the magnitude of perinatal risks is associated with the number and severity of neurological signs present in preschool children who were exposed to such risks (1). We also found that the presence of both perinatal risks and neurological signs was associated with long-term changes in circulating concentrations of molecules of the inflammatory process; the greater the magnitude of perinatal risks (asphyxia) and the greater the alteration of neurological signs, the greater the changes in inflammatory molecules. This suggests that chronic processes of even subclinical dysfunction may result in long-term manifestations of a low-grade inflammatory response (10).

Our next step was to focus on the study of inflammatory molecule profiles in various expressions of neurological disorders. We included (a) caregiver-perceived disorders (defined as risk detection of regulatory disturbances, through the DeGangi instrument); (b) vegetative-type autonomic disturbances (dysautonomias) and asymmetries, diagnosed through neurodevelopmental assessments performed by specialists; (c) cognitive developmental disturbances (through the Bailey instrument). Our objective was to evaluate whether the different expressions of neurological disorders show variations in their inflammatory molecule profiles or whether there is a common pattern.

Our results show that different neurological alterations, which are expressed as alterations of tone, asymmetries, pathological reflex responses, or cognitive impairment are reflected in different, even opposite, inflammatory profiles, suggesting that the detectable molecular footprints associated with function disorders respond to complex dynamics of molecular adjustment to challenges, damage and sequelae. In the case of high risk to develop regulatory disturbances, we detected what we called a state of hyper-resolution. We propose a theoretical model describing possible scenarios for inflammatory outcomes linked with chronic challenges due to functional disorders.

## Materials and methods

This observational, descriptive, cross-sectional, partially retrospective study was performed in preschoolers born in Mexico City in the period from March 2014 to December 2016, who had neurodevelopmental assessments and neurological diagnoses. The retrospective part consisted of obtaining data on dysautonomia diagnosed during the first year of life. All children were part of the cohorts of the Neurodevelopmental Research Center of the National Institute of Pediatrics. Sampling and laboratory analyses were performed at the National Institute of Cardiology. For children under 6 months of age the neurodevelopmental check-up was performed monthly, after this age and up to one and a half years of age it was every 2 months and for consecutive ages, every 4 months. The Bayley III scale was used for child development assessments. This scale includes five domains: cognitive, motor (gross and fine), language (expressive and receptive), social-emotional, and adaptive. Clinical assessment by experts included detection of signs and symptoms dependent on the type of muscle tone and its severity; maturation or alteration of reflexes; presence of dysautonomic signs; organization of developmental patterns in sitting and displacement; and appearance and/or disorganization or organization of pyramidal activity. Parents or guardians completed the DeGangi instrument for early detection of risk for developing regulatory disorders. The DeGangi instrument yields scores related to the risk of developing regulatory disorders in several areas including. (1) Self-regulation, (2) attention, (3) sleep, (4) eating, (5) dressing, bathing and touch, (6) movement, (7) hearing, language and sound, (8) vision, and (9) attachment-emotional functioning. We divided the total scores into tertiles to define the relative risk of developing these disorders as low, medium, and high. All parents or legal guardians of the children signed a letter of informed assent explained in detail. The research and ethics committees of the National Institute of Cardiology approved the research protocol (#17-1032). It was also approved by the Research and Research Ethics Committees of the National Institute of Pediatrics (registration numbers 060/2011; 088/2013; 75/2014; 059/2014; and 047/2015).

The gestational age of all participating infants was term, between 37 and 42 weeks. All were eutrophic with an average weight of 3,245 g (2,950–3,540). The 15 infants with asphyxia required invasive ventilation, with Apgar less than 7 in the first minute of life. The remaining 12 infants with Apgar greater than 7 did not require invasive ventilation or advanced reanimation measures. The results correspond to the last assessment (regulatory, neurological, and cognitive), made on average at 34.7 months of age (see Table 1 of sociodemographic data); the values of inflammatory molecules were taken from the measurement closest to that assessment, which in no case was greater than one month. The accepted protocol included a total of 2 blood samples, taken with a 7-month interval.

**Table 1:** Sociodemographic and clinical characteristics of the sample.

	*N* or *χ*	% or SD
**Gender**		
Female	11	40.7
Male	16	59.3
**Childs’ age, months. *χ* (SD)**	34.7	(12.7)
**Mothers’ age. *χ* (SD)**	24.3	(7.26)
**Fathers’ age. *χ* (SD)**	28.9	(9.03)
**Mothers’ occupation**		
Home	23	85.2
Employee	2	7.4
Student	2	7.4
**Fathers’ occupation**		
Formal employee	6	22.2
Informal employee or independent worker	13	48.1
Underemployed	5	18.5
Student	3	11.1
**Fathers’ schooling, years. *X* (SD)**	11.27	(3.28)
**Level**		
Basic	3	11.1
Middle	12	44.4
Superior	12	44.4
**Mothers’ schooling, years. *X* (SD)**	11.16	(3.13)
**Level**		
Basic	2	7.4
Middle	15	55.6
Superior	10	37
**Initial diagnosis**		
Perinatal asphyxia	15	55.6
Congenital hypothyroidism	4	14.8
Mixed encephalopathy	5	18.5
Congenital toxoplasmosis	2	7.4
Congenital heart disease	1	3.7
**Regulatory risk**		
Low	10	37.0
Medium	7	25.9
High	10	37.0
**Dysautonomia**		
Absent	12	44.4
Present	15	56.6
**Asymmetry**		
Absent	19	70.4
Present	8	29.6
**Cognitive impairment**		
Absent	12	60.0
Present	8	40.0

### Inclusion criteria

We included all the children within the required ages (4–5 years) who had more than three neurodevelopmental assessments, who were active in the follow-up cohorts for the following perinatal risks: asphyxia, congenital hypothyroidism, mixed encephalopathy, congenital toxoplasmosis, and congenital heart disease (the perinatal risks are described in the table as “initial diagnosis”). The parents had signed a letter of informed consent. This letter included information on the number of blood samples required, type of testing, and use of the data. The document made it clear that participation was voluntary and that they could withdraw from the study at any time without consequence.

### Exclusion criteria

Children with a genetic syndrome, children with cerebral palsy, convulsive syndrome or chronic disease that could cause inflammation (cancer, asthma, allergies). Lack of blood samples or complete data.

### Collection of blood samples

We obtained 5–10 ml of peripheral blood in each collection, separated the serum, and made aliquots that were stored at −70°C. We asked the caregivers to bring the children without having consumed chocolate or coffee (because they are neurostimulants) during the previous 24 h; they were also asked to have only a glass of milk and half a portion of fruit for breakfast, at least 4 h before taking the sample. If an acute illness was diagnosed at the time of the study, the appointment was postponed for 14 days. We were able to obtain blood samples only from 27 children.

We analyzed the samples on a multiplex system on a Luminex MAGPIX (Luminex Corporation) using Affymetrix Human Inflammation Panel (20 plex) by eBioscience, following the manufacturer's instructions. The analytes and their detection ranges were: GM-CSF (18–73,300 pg/ml), IFN-gamma (11–11,075 pg/ml), TNF-alpha (6.98–28,600 pg/ml), IL-12p70 (6.67–6.825 pg/ml), IL-17A (2.27–9,300 pg/ml), IL-1beta (2.09–8,550 pg/ml), IL-6 (11–10,850 pg/ml), sICAM-1 (212–870,200 pg/ml), E Selectin (250–255,500 pg/ml), P-Selectin (1,233–5,051,600 pg/ml), IFN-alpha (0.49–2,000 pg/ml), IL-1 alpha (0.55–2,250 pg/ml), IL-8 (2.47–10,100 pg/ml), IP-10 (1.25–5,100 pg/ml), MCP-1 (1.15–4,700 pg/ml), MIP-1alpha (1.86–1,900 pg/ml), MIP-1beta (4.22–17,300 pg/ml), IL-4 (11–45,200 pg/ml), IL-13 (2.59–10,600 pg/ml), and IL-10 (2.49–10,200 pg/ml).

We used ELISA kits to determine Resolvin D1 (RvD1, Cayman Chemical, assay range 3.3–2,000 pg/ml and sensitivity of approximately 15 pg/ml), Lipoxin A4 (LXA4, Cloud-Clone Corp. CEB452Ge, detection range 493.8–40,000 pg/ml, minimum detectable is usually less than 166.1 pg/ml), leukotriene B4 (LTB4, R&D Systems KGE006B, sensitivity 10.9 and assay range 10.3–2,500 pg/ml), and Annexin A1 (Cayman Chemical, 501550, range 0.20–20 ng/ml, sensitivity 0.2 ng/ml and lower limit of detection 0.18 ng/ml), in all cases following the manufacturer's instructions.

All methods had the required quality controls. In the results report we only include molecules with statistical significance.

### Statistical analysis

We transformed the statistical data on the concentrations of inflammatory molecules into standardized values with a mean of 0 and a standard deviation of 1 to express them as *Z*-scores. Since we found no differences by age and sex, no adjustments for these variables were necessary. All children included in the study had long-term follow-up. In our cohort all children had nutritional counseling and were within adequate parameters in weight and height for age, therefore we did not consider feeding, weight and height as confounding variables. Neurological sign variables were compared with standardized values of inflammatory molecules. Means of more than two groups were compared by analysis of variance (ANOVA) and a Tukey–Kramer *post hoc* test. If the data did not meet the parameters of homogeneity of variance and normal distribution, nonparametric statistics were performed with the Wilcoxon test. Because of the sample size and the dispersion of the groups, we considered the differences to be significant when the probability of error was *p* < 0.05 and marginally significant when it was between 0.05 and <0.1. In the latter case, we further performed univariate comparisons to assess significance.

## Results

**Figure 1:**
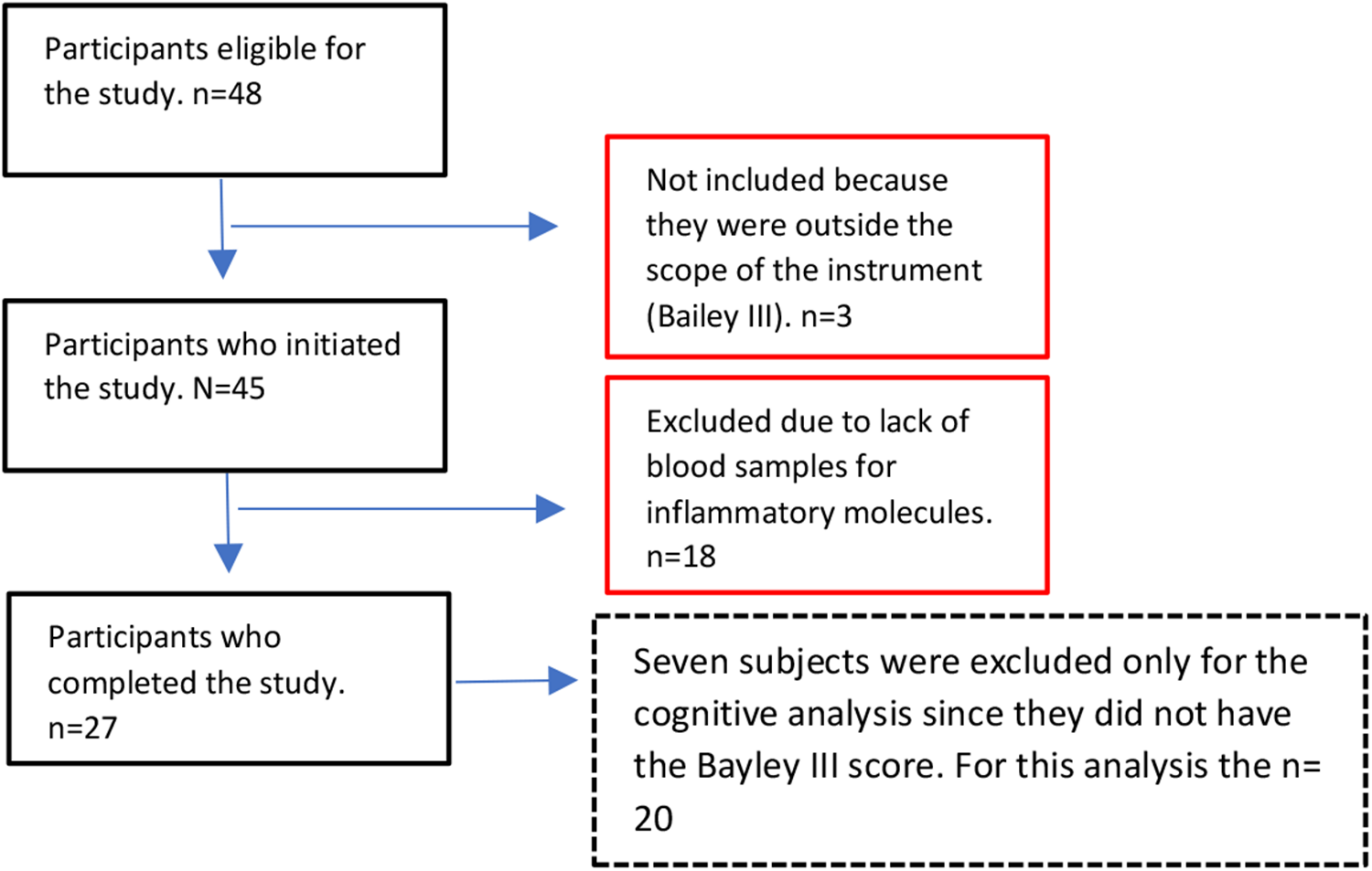
Flow chart illustrating the study sample selection.

At the time of data collection, the whole cohort with neurodevelopmental assessment included 45 children (20 Female, 25 Male). For this study we selected the 27 children that had a complete assessment of the inflammatory molecules. For the cognitive analysis seven subjects were excluded because they did not have the Bayley III score (*n* = 20 for cognitive analysis) (Figure 1).

### Inflammatory molecules and risk for regulatory disorders

**Figure 2:**
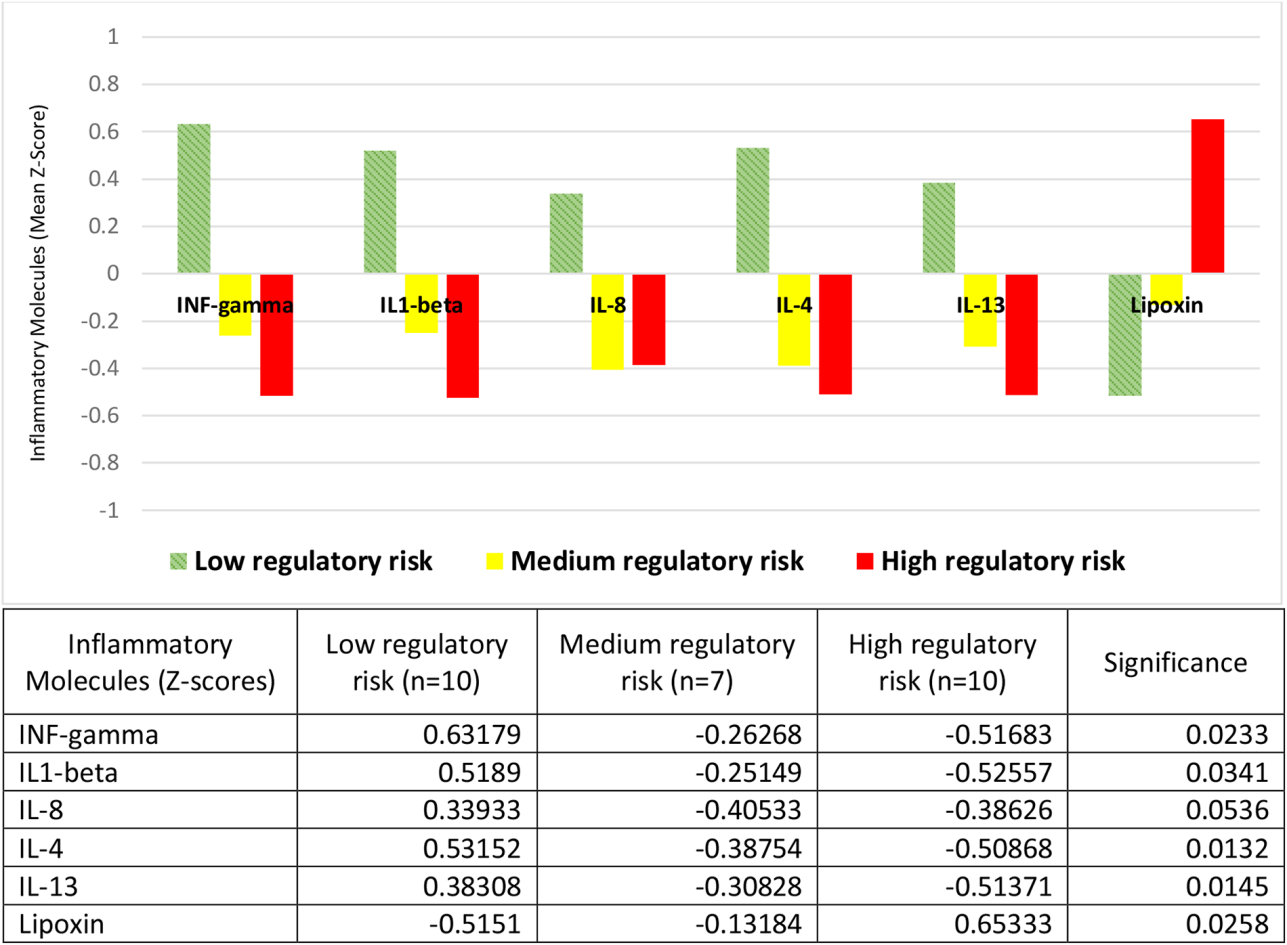
Inflammatory molecules and risk of regulatory disorders. Children at low regulatory risk had positive *Z*-scores for the mean concentrations of the proinflammatory molecules INF-gamma, IL1-beta and IL-8, and the anti-inflammatory molecules IL-4 and IL-13; and negative *Z*-scores for the pro-resolving molecule lipoxin. The *Z*-scores of the other two groups (medium and high regulatory risk) had the opposite sign, except for lipoxin in the medium-risk group, which had negative *Z*-scores like the low-risk children. The table expresses the complete numerical data and the significances across the groups.

The risks for regulatory disorders were measured with the De Gangi instrument, which assesses physiological data on sleep, feeding, reactivity to sensory stimuli, social and responsive behavior, according to the perception of parents or primary caregivers. We found the concentrations of inflammatory protein molecules to be inversely related to the risk of developing a regulatory disorder; both pro-inflammatory (INF-gamma, IL1-beta, and IL-8) and anti-inflammatory (IL-4 and IL-13) (Figure 2). Thus, in children at high risk for a regulatory disorder we found the lowest mean values of these cytokines (all with negative *Z*-scores), and the highest values of the pre-resolutive lipoxin (with positive *Z*-scores). At the opposite extreme, with a mirror image, in children at low risk we found the highest mean concentrations of the same cytokines (all with positive *Z*-scores), and the lowest for lipoxin (with negative *Z*-scores). Medium-risk children presented cytokine averages with negative *Z*-scores, in intermediate concentrations, although closer to those of high-risk children. Lipoxin *Z*-scores in this group were also negative, with concentrations closer to those of children at low risk (Figure 2).

### Inflammatory molecules and neurological alterations

**Figure 3:**
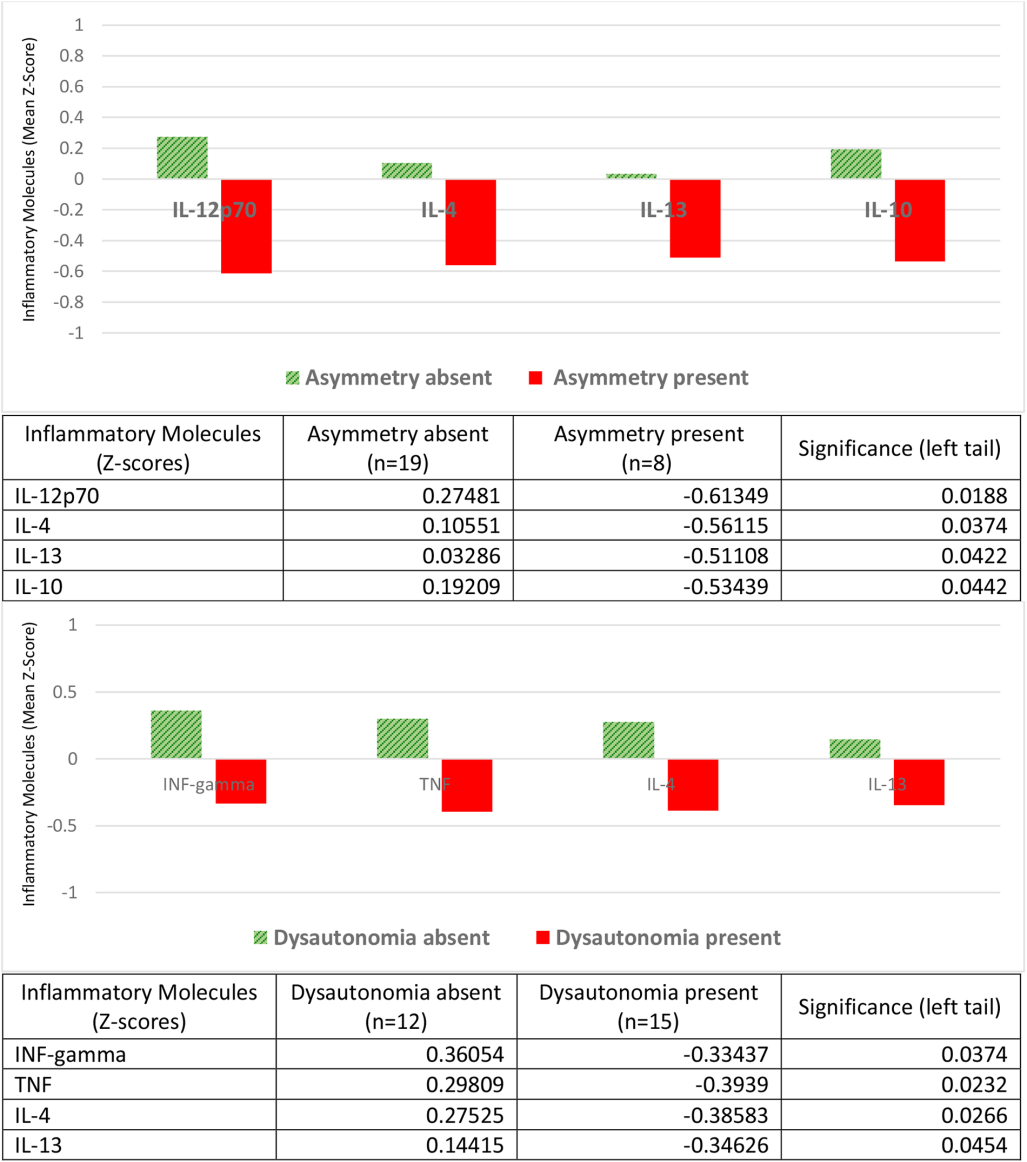
Inflammatory molecules and neurological disorders: asymmetry and dysautonomia. Children with neurological alterations had negative *Z*-scores for mean concentrations of the proinflammatory molecules IL-12p70 (asymmetry), or IFN-gamma and TNF (dysautonomia); they also had negative *Z*-scores for the anti-inflammatory molecules IL-4 and IL-13 (asymmetry and dysautonomia) and IL-10 (asymmetry). Children without neurological alterations had positive *Z*-scores for these cytokines. Tables express complete numerical data and between-group significances.

The neurological alterations were assessed based on a clinical diagnosis established by specialists. These include asymmetry and dysautonomia (Figure 3). The latter encompasses autonomic and motor disorganization and behavioral regulatory disorders. Both neurological alterations were associated with lower levels of cytokines (negative *Z*-scores) compared to children who did not manifest them (positive *Z*-scores). The pro-inflammatory cytokines found in lower concentrations were IL-12p70 in the presence of asymmetry, and IFN-gamma and TNF in the presence of dysautonomia. The anti-inflammatory ones were in both cases IL-4 and IL-13, in addition to IL-10 in asymmetry (Figure 3).

### Inflammatory molecules and alterations in child cognitive development

**Figure 4:**
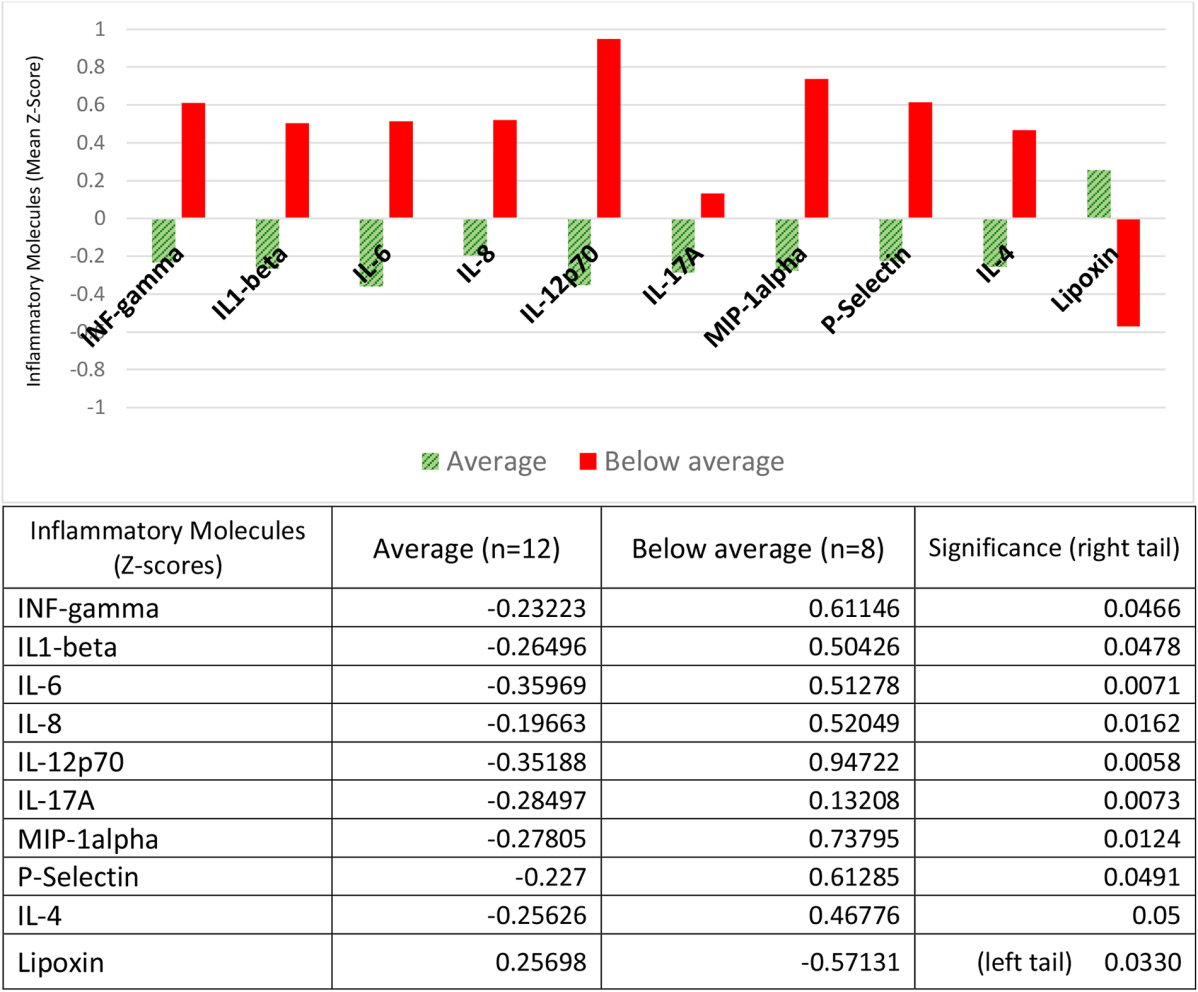
Inflammatory molecules and alterations in child cognitive development. Children with cognitive impairment (below average) had positive *Z*-scores for mean concentrations of the proinflammatory molecules IL1-beta, IL-6, IL-8, IL-12p70, IL-17A, MIP-1-alpha, INF-gamma and P-Selectin, and the anti-inflammatory cytokine IL-4; children with average (normal) cognitive development had negative *Z*-scores for all these cytokines. Pro-resolving lipoxin had negative *Z*-scores in the cognitively impaired group and positive *Z*-scores in the cognitively normal group. The table expresses the complete numerical data and the significances between the groups.

We assessed child's development with the Bayley III instrument. Of the five domains, we only report on cognitive development because only in this domain did we find statistically significant differences between the groups. Contrary to what was found for children with neurological alterations and risk of regulation disorders, children with below average cognitive development (cognitive impairment), had a higher concentration of 8 pro-inflammatory molecules (IL1-b, IL-6, IL-8, IL-12p70, IL-17, MIP1, INF-g and P-Selectin), and one anti-inflammatory cytokine (IL-4,) all with positive *Z*-scores, compared to children with average (normal) cognitive development, who had negative *Z*-scores for all of them. The pro-resolutive lipoxin had an inverse behavior: a lower concentration (with negative *Z*-scores) in the cognitively impaired group, and a higher concentration (with positive *Z*-scores) in the cognitively normal group (Figure 4).

### Dynamics model of the inflammatory response: Persistent systemic stress and hyper-resolution

**Figure 5:**
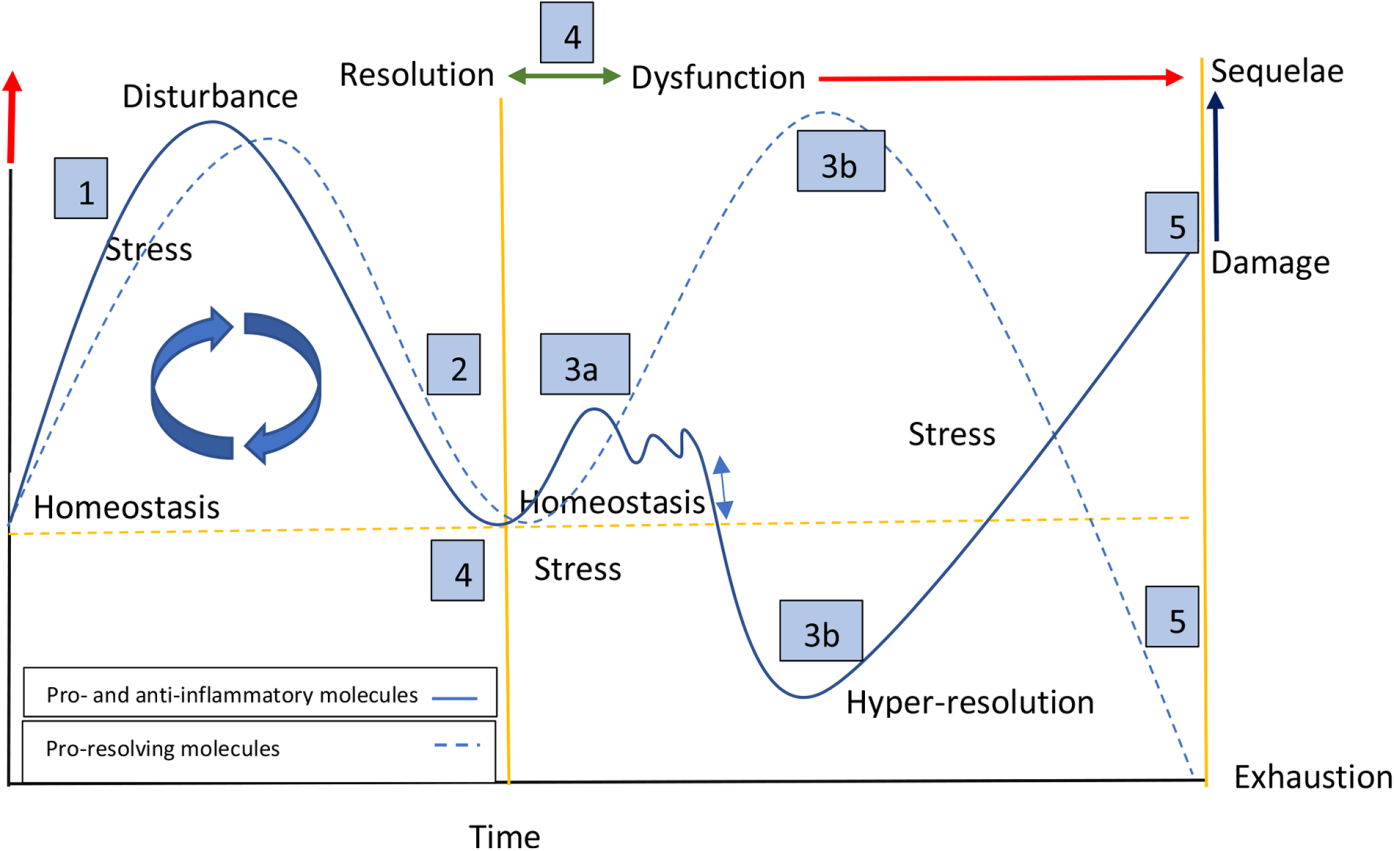
Dynamic model of the inflammatory response: persistent systemic stress and hyper-resolution. **Left panel**: a typical acute response starting from homeostatic parameters. Triggered by cellular stress, homeostasis is disrupted and (1) pro-inflammatory/anti-inflammatory, and (2) pro-resolving responses are subsequently activated. **Right panel**: persistent tissue dysfunction leads to two possible pathways: (3a) sustained, low-grade, fluctuating, and parallel pro-inflammatory and anti-inflammatory responses, with no or low pro-resolving response; (3b) depressed pro-inflammatory and anti-inflammatory responses, in some cases accompanied by elevated concentrations of pro-resolving molecules (hyper-resolution). (4) If the dysfunction resolves, the system would return to a homeostatic state (bidirectional green arrow, and crossing of yellow lines). (5) If dysfunction continues (red arrow), a point of no return may be reached along with long-term inflammation, depletion of pro-resolving molecules, damage, and sequelae.

Based on our findings and those of others, we developed a theoretical model that proposes that alterations in function would not be associated exclusively with increased concentrations of proinflammatory molecules, but would be embedded in a complex dynamic of molecular adjustment in response to challenges, damage and sequelae (Figure 5). The left panel of the graph shows a typical acute response starting from a point defined by individual conditions within homeostatic parameters (11). In the face of different challenges that provoke cellular stress, the pro-inflammatory response is activated, with subsequent anti-inflammatory and pro-resolving responses seeking a return to homeostasis (12). The right panel represents what we postulate would happen in the face of persistent tissue dysfunction caused by previous damage (13). In this case, although the acute damage has disappeared, the chronic cellular stress caused by a primary or secondary dysfunctional system would result in two possible pathways: (a) a sustained but fluctuating pro-inflammatory response, typically of low grade, accompanied by the respective anti-inflammatory response of similar intensity, with a notable absence of a pro-resolving response. In our data we found this pattern associated with cognitive stress; (b) a depressed response characterized by low concentrations of pro- and anti-inflammatory molecules and, in some cases (high risk of regulatory disorders), with a clear predominance of pro-resolving molecules, a phenomenon we call hyper-resolution. We found this pattern associated with regulation disorders and motor asymmetries. Theoretically, if the stressful stimulus disappears (the dysfunctionality), the system would return to a homeostatic state (14). If this does not happen, the hyper-resolution state could be exhausted giving way to long-term inflammatory disorders that could reach a point of no return (15) (Figure 5).

## Discussion

The growth and maturation of the organism occurs from the internal and external stimuli to which the individual is subjected (16). The organization of growth is affected not only by genetics but also by environmental stressors (17), which together can create in the individual an imprint of damage at different levels. High-risk pregnancies and perinatal complications can generate acute damage that is associated with sequelae in the neurodevelopment of the infant, caused by direct lesions in neurons and neural progenitor cells (18). The long-term extension of these sequelae opens the possibility that in persistent neurodevelopmental disorders causing tissue dysfunction, beyond the inducing agents, underlying inflammatory phenomena may determine the perpetuation of a state of low-grade inflammation or parainflammation (19). Inflammation can also generate indirect injury through the activation of microglia and astrocytes that can release cytokines and reactive oxygen species (20). In animal models it has been shown that the inflammatory response increases the risk of altering brain structures that can undergo changes not only in shape, consistency, and size, but also in gene expression and protein production, thus modifying the response to external and internal stimuli (21).

In agreement with the postulate that chronic dysfunction may be associated with inflammatory modifications, we found that the profiles of molecules of the inflammatory process are different in groups with persistent neurodevelopmental alterations than in those without. Moreover, the distribution and concentration of the molecules were different for each type of dysfunction. Our results show that it is possible to detect an immunological molecular footprint associated with chronic neurological dysfunction, with two opposed patterns. In the presence of cognitive impairment, a proinflammatory response predominated, with a mild anti-inflammatory response, accompanied by a depressed resolution response. On the other side, the mediator profiles in children without cognitive dysfunction, but with dysautonomia, functional asymmetry or risk of regulatory disorders were characterized by diminished proinflammatory and antiinflammatory responses. In children at risk for regulatory disorders, inflammatory cytokine concentrations were lower and pro-resolutive mediators were higher as the risk for regulatory disorders increased. In the presence of a high risk of regulatory disorders, we detected a strong pro-resolutive response which we termed hyper-resolution. We suggest that this hyper resolutive state would be a late consequence of sustained inflammatory stimulus (see Figure 5).

We hypothesize that neurodevelopmental dysfunctions could generate stress, local or systemic, sufficient to alter the individual's inflammatory responses. The persistent alteration of the nervous system would result in permanent challenges to homeostasis that would provoke cellular stress responses (22). These responses would depend on the brain structure, chronicity and level of consciousness (and therefore emotional involvement) of the condition. The conjunction of these factors would explain why each alteration is accompanied by a different molecular profile. This hypothesis would also explain why the molecular profile of cognitive impairments (measured with the Bayley instrument) is opposite to that of other impairments: neurodevelopment is continuous and therefore the child faces changing challenges in a permanent process of stress that can be considered acute.

Brain plasticity allows functional adaptation after damage, which opens the opportunity for early intervention in children with a history of damage or at risk of developing neurodevelopmental alterations (23). Our cohort was subjected to stimulation and early interventions from the first months of life and, in all cases, the children were independent and attended day care centers and schools where they are qualified within normal parameters. However, in the follow-up we found that early acute damage secondary to high perinatal risks can be associated with persistent dysfunctions in different areas. Especially, we have children with regulatory disturbances that do not seem to subside despite early interventions; specifically, motor functional asymmetry is difficult to reverse and is often perpetuated. Functional asymmetry is an early neurological sign of dissociation in neural development and growth, and can be affected by biological as well as social and parenting factors. The poor organization of children with functional asymmetry may be related to alterations at the level of neuronal migration and maturation (24). Regardless of the cause, we found that its presence was associated with alterations in inflammatory molecules.

Our data suggest that the fingerprint of change at the molecular level that is associated with chronicity of dysfunction may also be present in the disorganization of learning pathways in children with regulatory disorders. Regulation is a dynamic process that depends on developmental processes and biological and environmental factors, such as social participation. It can determine behaviors in daily life and in states of stress, with responses within accepted parameters, or excessive or attenuated (25). Children with adequate regulation are better able to build number learning, color differentiation, and letter structures than those with regulation disorders. The latter are related to disruptive temperaments at early ages, before one year of age, and alterations in certain areas of preschool learning from 4 years of age onwards (26).

Up to 20% of infants may present dysautonomia, a regulation disorder, in the first months of life, visible to parents by signs such as sleep disturbances, inconsolable crying and changes in skin color when crying; however, only a small group exceeds one year of age with these disorders (27), which could be related to the maturation of the reticular system. We postulate that the presence of dysautonomia after one year of age, would force the immune and neurological system to readapt, or to maintain a constant low-grade inflammation, depending on the resilience of the system. Children with features of regulatory disorder may develop increased anxiety and depression (27). We found no literature linking regulatory disturbances in children and immunological processes; it could be inferred that negative emotionality in children, described as the child's general propensity for distress, would be similar to neurosis in adults. In both cases we would interprate it as a state of susceptibility to stress, to stimuli of lower than normal intensity (28). However, our children with regulation disorders presented opposite molecular profiles to adults with depression, probably because in children under 5 years of age the chronicity is lower, and the inflammatory responsiveness is in a different state (see Figure 5).

The molecular profile of the children in our cohort with cognitive disorders has more similarities with that of depressed adults, in whom higher concentrations of proinflammatory cytokines are found than in the non-depressed. In fact, treatment is usually more effective in those with lower concentrations of proinflammatory molecules. Similarly, antidepressant treatments can generate a decrease in the concentration of proinflammatory molecules (29). The molecular profile of our children with cognitive disorders also resembles that of persons subjected to emotional stress during childhood (30). In them, there is a cortical/amygdalin neuronal sensitization to trauma, which causes the neuroimmunological response to the effector stimulus to be more intense and rapid (30). Abused children and adolescents of low socioeconomic status show higher concentrations of IL-6, and their monocytes and macrophages are relatively insensitive to inhibition by glucocorticoids, which play a key physiological role in the regulation of inflammation. Similar patterns are evident in youngsters raised in difficult family environments (31).

Experiments in mice have shown that high concentrations of IL-6 are sufficient to alter normal neurodevelopment (32). Inflammatory markers could function as predictors of neurodevelopment in children: higher concentrations of IL-1β and IL-6 at 6 months of age are significantly associated with a decrease of almost five points in Bayley III rated motor score at 2 years of age. In contrast, an elevation of Th2 cytokines, such as IL-4, is associated with an increase in cognitive score of almost 4 points (33). In agreement, our results show that children with higher concentrations of IL-1β and IL-6 (and other proinflammatory mediators) have lower Bayley III cognitive scores; however, our group of children with low cognitive status also has higher concentrations of IL-4, although lower concentrations of the resolving mediator lipoxin. Despite some differences, in both studies the molecules function as markers of dysfunctionality (33).

The relationship between circulating cytokines and CNS reorganization or dysfunction is becoming increasingly clear. Cytokines are crucial in brain function, neurodevelopment, neurogenesis, and neurotransmission; cytokine receptors are expressed on microglia, astrocytes, and neurons. In particular, interferon-γ, interleukins (IL)-1β, -6, -8, -10, -17A and tumor necrosis factor (TNF) are thought to play a role in the association between immunity and mental or physical health (34). IL-17A has been specifically implicated in sensory regulation in experiments in mice. The A subunit of its receptor is expressed in cortical neurons, including the Primary Somatosensory Cortex Disgranular Zone (S1DZ) (35).

Inflammation can modulate the structure, function, and development of the cortex in areas such as the hippocampus; it is present in hippocampal neurodegeneration and in impairment related to memory function (36). This raises the possibility that low-grade systemic inflammation, or parainflammation, could predict subclinical cognitive impairment in part through structural neural pathways, as demonstrated by our data in children undergoing Bayley III testing, cognitive area. Adequate organization in neurodevelopment is dependent not only on the proper growth of CNS structures, but also on the maturation of these structures, strongly linked to extrinsic and intrinsic stimuli. As part of the latter, molecules of the inflammatory process seem to play a crucial role.

Our results show that chronic disorders in organ function would not be associated exclusively with an increase in the concentrations of proinflammatory molecules, but would be immersed in a complex dynamic of molecular adjustment in response to various challenges, damage or sequelae. Thus, the observed molecular patterns could be variable, even opposite; it is possible to observe from a classical inflammatory response with few resolving molecules, to a hyper-resolving scenario that would limit the ability to fully establish an inflammatory response. A detailed mapping of immune/inflammatory response mediators in different states of chronic dysfunction is necessary to identify the moment of the dynamic process in which a particular subject's response is found, for a more specific and better targeted management.

Our children are cared for by a highly specialized multidisciplinary team, and have a detailed and carefully documented follow-up; however, our study has some limitations. From the original cohort of 45 children, we only had complete data on inflammatory molecules for 27 children. The analyses were performed on these, which meant that for some of the analyses we had a reduced number of children. Another limitation is methodological: the gold standard for determining lipid mediator concentrations is liquid chromatography coupled to mass spectrometry, and we used ELISA kits.

## Conclusion

Our work shows that neurodevelopmental disorders are associated with characteristic and variable molecular profiles of the inflammatory response depending on the type of disorder. They present a molecular fingerprint, whose pattern is opposite, specular, when comparing that of children with cognitive impairment with those who only manifest neurovegetative disorders. In the former, the balance leans toward an inflammatory response; in the latter, toward a resolutive response. In children at high risk of regulatory disorders, we found a strong resolutive response, which leads us to postulate that a sustained inflammatory stimulus could condition a hyperresolutive state.

## Data Availability

The raw data supporting the conclusions of this article will be made available by the authors, without undue reservation.
